# MAPPING RELIGIOUS AND SPIRITUAL CARE IN WISCONSIN LONG-TERM CARE: A DESCRIPTIVE STUDY

**DOI:** 10.1093/geroni/igae098.4238

**Published:** 2024-12-31

**Authors:** Kathryn Lyndes, Sarah McEvoy, Louis Arata

**Affiliations:** Saint Mary’s College, Notre Dame, IN, Notre Dame, Indiana, United States; Rush University Medical Center, Chicago, Illinois, United States; Saint Mary’s College, Notre Dame, Indiana, United States

## Abstract

The importance of religion and spirituality (R/S) for older adults is well-recognized (Pew Research Center, 2015); however, little is known about specific types of R/S care and who provides it across a range of long-term centers. The aim of this study was to expand on a pilot study that explored the feasibility of gathering details about R/S care of residents in long-term care centers in northern Indiana (Lyndes & Fitchett, 2021), and on a single Tennessee county study that enabled the researchers to hone the study design (Lyndes, McEvoy, & Arata, 2023). The current study in this abstract extended the sample to Wisconsin, employing a systematic selection of diverse residential care centers (including skilled nursing homes, assisted living, independent living, and senior housing), and developing a preliminary description of the types of services as well as the training and criteria of the staff and volunteers who provide them. Results indicate that the majority of multi-disciplinary staff who participated in this study identified R/S care as beneficial to the health and well-being of residents. Many centers rely on staff chaplains and/or volunteer community members, with a range of credentials and training such as Clinical Pastoral Education, to provide an array of R/S services, for example one-on-one visits and weekly worship. The data has important implications for allied healthcare teams and long-term care providers who deliver services to older adults, including the need for culturally competent training across faith traditions to meet the needs of diverse residents.

